# Can digital governance promote urban energy conservation and emission reduction? A quasi-natural experiment based on “National Pilot Policy of Information Benefiting the People” in China

**DOI:** 10.1371/journal.pone.0320007

**Published:** 2025-03-25

**Authors:** Shaohui Zou, Yingying Ji

**Affiliations:** School of Management, Xi’an University of Science and Technology, Xi’an, China; Zhejiang Shuren University, CHINA

## Abstract

The digital transformation of government is an important path to achieve the modernization of the national governance system and governance capacity. In recent years, the “National Pilot Policy of Information Benefiting the People” has become a key measure for big data to empower government governance, accelerate the improvement of public service levels and equalization of public services. This paper adopts the difference-in-difference method, takes the “National Pilot Policy of Information Benefiting the People” implemented in my country in 2014 as a quasi-natural experiment of digital governance, and explores the effectiveness of government digital governance measures on urban energy conservation and emission reduction and its potential transmission mechanism based on panel data of 283 cities in China from 2006 to 2021. The study found that the construction of digital government has a positive role in promoting urban energy conservation and emission reduction, and this positive impact is mainly achieved by promoting green technology innovation and strengthening environmental regulation; there are differences in the impact of digital governance on urban energy conservation and emission reduction among cities of different regions, different scales, and different resource endowments. This paper studies the energy conservation and emission reduction effect of government governance from the perspective of digital transformation, and provides important empirical inspiration for the sustainable development of cities under the dual carbon goals and the formulation of energy conservation and emission reduction action strategies.

## 1. Introduction

In recent years, extreme weather and climate events such as rainstorms, floods, heat waves and droughts have occurred frequently in many parts of the world, causing huge economic losses [1]. The World Meteorological Organization stated that such events have become the new normal, and climate change caused by greenhouse gas emissions is the main cause. Therefore, promoting greenhouse gas emissions, mainly carbon dioxide, and promoting low-carbon transformation have become a common concern of governments around the world [2]. China’s total energy consumption has increased from 571 million tons of standard coal in 1978 to 5.41 billion tons of standard coal in 2022. In particular, the large-scale consumption of traditional fossil energy has led to a continuous increase in total carbon dioxide emissions, and has also caused serious energy-related environmental problems, such as air pollution and greenhouse effect [3]. The environmental problems caused by energy consumption have not only seriously hindered the green transformation and development of China’s economy [4], but also caused huge economic losses to China [5]. In response to the environmental crisis and climate change, the Chinese government has set ambitious goals of achieving carbon peak by 2030 and carbon neutrality by 2060. This is not only an important path to achieve sustainable development, but also an important driving force for my country's economic structure transformation and energy structure optimization [6]. As an important engine for national economic development, cities are the main focus of regional energy consumption and carbon emissions [7]. Carbon emissions in urban areas in China account for about 80%. This data reveals the importance and urgency of urban decarbonization [8]. Therefore, effectively controlling urban energy consumption and reducing carbon emissions has become an urgent task facing the Chinese government. In this context, the government, as a policy maker, policy implementer and resource allocator, plays an irreplaceable role in promoting urban energy conservation and emission reduction and achieving sustainable development goals [9].

With the in-depth practice and development of digital technology, governments of various countries have actively innovated management concepts and governance models, and digital transformation has become an important direction of reform [10]. Digital governance, an emerging government governance model, has become a hot topic of discussion in academia. Some scholars point out that the digital transformation of the government plays a key role in improving the quality of government services [11], promoting collaborative management [12], and assisting scientific decision-making [13]. It is a strong driving force for the high-quality development of the digital economy, the optimization of the business environment, and the convenience of business and entrepreneurship for enterprises and the public [14]. At present, scholars have conducted in-depth research on various factors affecting urban energy conservation and emission reduction, including the promotion and use of clean energy [15], the optimization of energy structure [16], the upgrading of industrial structure [17] and environmental regulation [18]. Data elements penetrate and empower traditional elements, and reconstruct them in combination, thereby promoting technological change and efficiency change [19]. As a new type of production factor, digital technology has been deeply applied and practiced in the fields of economic growth [20], rural revitalization [21], agricultural innovation [22], and ecological and environmental protection [23], profoundly changing the traditional production model and industrial structure [24]. The application of digital technology has promoted the digital transformation of various industries, reduced the cost of renewable energy, and accelerated the transition to sustainable energy [25]. A large number of studies have shown that digital technology can achieve the coordinated development of economic growth and carbon emission reduction [26]. Digital development, based on digital technology, also plays an important role in promoting energy efficiency and promoting low-carbon development. On the one hand, digitalization has accelerated the elimination of backward production capacity in the fields of production and operation management, reduced energy loss [27], promoted the development of the clean energy industry, and optimized the energy structure [28]. On the other hand, digitalization has accelerated the pace of urban transformation to a low-carbon economy with its technological spillover effect [29]. The scale effect of the digital economy has also prompted surrounding areas to take action to reduce energy consumption intensity and improve energy efficiency [30]. However, improving urban energy efficiency and reducing carbon emissions largely depends on the government's governance capabilities [31]. Digital technology and its widespread application provide new opportunities for the government to innovate management concepts and governance models. Countries around the world have also actively conducted research on the relationship between the digitalization of government governance models and environmental sustainability [32].

There have been extensive studies on the impact of digital technology and digitalization on energy conservation and emission reduction, but there is still a lack of research on how digital governance affects the government's ability to manage energy and carbon emissions. Energy and environmental constraints restrict the high-quality development of China’s economy, and energy conservation and emission reduction are inevitable choices. So, in the context of the deepening digital transformation of the public sector, can digital governance affect urban energy conservation and emission reduction? What is the mechanism behind it? Exploring the above issues will help alleviate the urgent problems of urban development such as energy waste and environmental degradation, and will be of great significance to improving urban modernization governance and achieving sustainable urban development. Therefore, this study uses the “National Pilot Policy of Information Benefiting the People (NPIB)” as a quasi-natural experiment to study digital governance [33]. The policy covers 80 cities in 31 provinces across the country, of which 71 cities were selected as the experimental group, and 9 cities were excluded because of serious data missing. The article selects panel data from 283 cities in China between 2006 and 2021. Based on the difference-in-difference method, it aims to explore the impact of digital governance on urban energy conservation and emission reduction, and analyze its potential transmission mechanism.

The contributions of this paper are as follows: On the one hand, this paper expands the relevant research on the factors affecting energy conservation and emission reduction from the perspective of digital governance. Existing research on energy conservation and emission reduction focuses on the impact of digital technology [34], digital economy [35], enterprise digital transformation [36], technological innovation [37] or industrial structure upgrading [38] on energy conservation and emission reduction, but pays less attention to the energy conservation and emission reduction effects of digitalization of government, an important market entity, and provides empirical guidance for policy makers of energy conservation and emission reduction. On the other hand, this study focuses on the environmental effects of digital governance and enriches the empirical research framework of digital governance. Existing research mainly focuses on the economic and social effects of digital government construction. This study provides strong empirical evidence for the environmental effects and mechanisms of digital governance.

## 2. Research hypothesis

### 2.1 Digital governance and urban energy conservation and emission reduction

As a key force in scientific and technological innovation and green transformation, digital technology promotes the coordinated development of pollution control and carbon emission reduction [39]. First, through information technology, data sharing and integration among government departments can be achieved, information barriers between departments can be broken, efficient data circulation and centralized resource management can be achieved, and the overall governance level of the government can be improved [40]. With the help of online approval systems, the process is simplified, the time cost and systematized transaction cost of the public and enterprises are reduced [41], and the efficiency of resource allocation is optimized, thereby reducing energy waste and urban carbon emissions. Secondly, digital technology provides support for the optimization of government environmental supervision models. Digital technologies such as big data and cloud computing can provide technical support for the government to monitor urban energy consumption, pollution emissions, environmental carrying capacity and other data in real time, thereby improving the accuracy and effectiveness of government environmental supervision, enhancing the government's environmental supervision capabilities, and reducing energy waste and environmental pollution [42]. Finally, the improvement of policy transparency and the expansion of public participation channels have encouraged market players and the public to actively participate in energy conservation and emission reduction actions, and promoted the use of renewable energy and the innovative development of environmental protection technologies. By promoting green practices such as online office and online meetings, digitalization provides important support for achieving low-carbon development, optimizing the use of public resources, and promoting sustainable urban development [43]. It can be seen that digital technology empowering government governance is a possible path to promote urban energy conservation and emission reduction and promote urban green and low-carbon transformation.

Based on this, a hypothesis (H1) is proposed: digital governance can promote urban energy conservation and emission reduction.

### 2.2 Mechanism of energy-saving effect of digital governance: green technology innovation

The implementation of the NPIB policy guides public and private capital to the green technology field, encourages the research and development and application of energy-saving, environmentally friendly and efficient technologies and products, provides policy support, financial guarantees and market environment for green technology innovation, stimulates innovation vitality and promotes sustainable urban development. From the perspective of innovation incentives, digital governance reduces the uncertainty and cost of green technology innovation by creating a carbon trading platform and a green financial service network, enhances the transmission effect of policy incentives and the market demand for green technology innovation. Green technology innovation achieves a win-win situation of economic growth and environmental protection by introducing efficient and low-emission technical means. On the one hand, green technology reduces energy waste and unnecessary consumption by optimizing energy use and improving energy conversion efficiency [44]. For example, the widespread application of smart grids, energy efficiency management systems and efficient lighting technologies makes energy distribution more accurate and intelligent, thereby effectively improving the overall efficiency of energy use [13]. On the other hand, green technology innovation promotes the optimization and upgrading of energy consumption structure by exerting technology promotion effect and spillover effect [45]. By developing and applying environmentally friendly technologies, we can promote energy supply-side reform, reduce dependence on coal consumption under the guidance of renewable energy technology, and promote the energy consumption structure to develop towards energy-saving and consumption-reducing mode [46].

Based on this, a hypothesis (H2) is proposed: digital governance improves urban energy efficiency by promoting green technology innovation.

### 2.3 Mechanism of the emission reduction effect of digital governance: environmental regulation

Government digital governance optimizes the implementation of environmental policies by enhancing the accuracy and transparency of environmental regulation. According to existing research, digital governance uses technologies such as big data and artificial intelligence to promote the real-time and precision of environmental supervision, enabling the government to conduct more comprehensive and dynamic monitoring of corporate emissions, pollution sources and environmental quality [47]. Specifically, digital platforms can collect and analyze environmental data in real time, helping the government to promptly detect excessive emissions and environmental violations [48], thereby enhancing the deterrent effect of environmental regulation and policy enforcement. Secondly, environmental regulation promotes the innovation and application of low-carbon technology and clean energy through policy guidance and financial support. In this process, the government reduces the innovation cost of green technology through green subsidies, tax exemptions and other measures, thereby accelerating the promotion and application of low-carbon technology and further reducing carbon emissions [49]. In addition, environmental regulation has promoted the socialization and institutionalization of carbon emission management by enhancing the environmental awareness of enterprises and society, forming a broader green development atmosphere [50]. Environmental regulation provides institutional guarantees for the realization of carbon emission reduction goals by strengthening policy constraints and incentive mechanisms.

Based on this, a hypothesis (H3) is proposed: digital governance reduces urban carbon emission intensity by strengthening environmental regulation.

**Fig 1:**
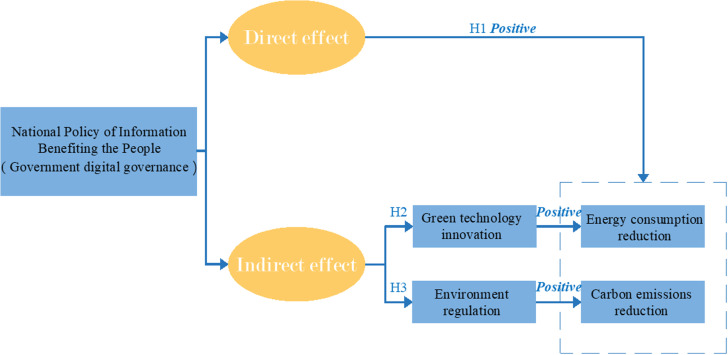
Impact mechanism.

Fig 1 shows the research framework of this paper.

## 3. Study design

### 3.1 Model

This paper studies the impact of digital governance on urban energy conservation and emission reduction. The NPIB policy implemented in 2014 is used as a quasi-natural experiment. The city-level differences and time-level differences of the research objects before and after the policy implementation are controlled, and DID model is used for empirical testing. The specific model construction is as follows:


$$\ln Energy_{it}=\alpha +\beta Treat_{it}\times Post_{it}+\gamma X_{it}+Year_{t}+City_{i}+\xi_{it}$$ (1)

$$\ln CO2_{it}=\alpha_{1}+\beta_{1}Treat_{it}\times Post_{it}+\gamma_{1}X_{it}+Year_{t}+City_{i}+\xi_{it}$$ (2)

Among them, the explained variable $\ln Energy_{it}$ is the energy efficiency of city *i* in period *t*, and the explained variable $\ln CO2_{\text{it}}$ is the carbon emission intensity of city *i* in year *t*, the above two variables are used to characterize urban energy conservation and emission reduction; $Treat_{it}$ is a policy dummy variable, which takes the value of 1 if the city participates in the NPIB policy, and takes the value of 0 if it does not participate; $Post_{it}$ is a time dummy variable, that is, after participating the NPIB policy, *Year* takes the value of 1, otherwise it takes the value of 0; the interaction coefficients *β* and $\beta_{1}$ reflect the impact of the digital government efficiency brought by the information benefiting the people pilot project on urban energy conservation and emission reduction, which are the two estimated coefficients that this paper mainly focuses on. This paper adds fixed effects and control variables to the regression model. Among them, $City_{i}$ is the city fixed effect, $Year_{t}$ is the time fixed effect, which is used to absorb the interfering factors that do not change with the city and time in the regression model, and to a certain extent alleviate the problem of omitted variables. $X_{it}$ represents other control variables, representing other factors affecting urban energy conservation and emission reduction except the NPIB policy. Referring to existing research, this paper selects the following control variables: total urban population (lnPOP), urban digital development level (DE), industrial structure upgrading (lnAIS), science and technology education level (lnSTE), green technology innovation (GTP) and environmental regulation (lnER). Referring to the study of Ma et al. [51], this paper uses the results calculated by the super-efficiency SBM model taking into account non-expected output to measure the level of urban green technology innovation.

### 3.2 Data source

The original data of each variable indicator in this paper are all from the “China City Statistical Yearbook”, “China Regional Economic Statistical Yearbook”, “China Statistical Yearbook” and the statistical yearbooks of various provinces and cities from 2006 to 2021. Based on the original data, this paper conducts the following processing: (1) Considering the availability and consistency of the control variable data, we exclude samples with serious data missing. (2) Linear interpolation is performed to supplement some missing data. Finally, the balanced panel data of 283 cities in China from 2006 to 2021 were obtained as the research sample.

### 3.3 Descriptive statistics

The descriptive statistical characteristics of the variables are shown in Table 1. Among them, the mean value of Treat is 0.237, indicating that about 23.7% of cities have entered the NPIB policy; the mean value of Post is 0.5, indicating that about half of the observations are after the policy implementation. The standard deviations of DE and lnER are relatively high, and there are large differences in the digital development levels and environmental regulation intensity of different cities.

**Table 1:** Descriptive statistics of variables.

Variable	Variable Description	Obs	Mean	SD
**ln*Energy***	Logarithm of urban energy efficiency	4528	2.529	0.786
**ln*CO2***	Logarithm of urban carbon emission intensity	4528	-8.423	0.731
***Treat***	Cities participating in digital governance are assigned a value of 1, otherwise 0	4528	0.251	0.434
***Post***	Time dummy variable, 0 before policy implementation and 1 after implementation	4528	0.500	0.500
**ln*POP***	The logarithm of the total urban population	4528	5.873	0.692
***DE***	Urban digital development level	4528	0.271	1.590
**ln*AIS***	Logarithm of industrial structure upgrading	4528	-0.145	0.481
**ln*STE***	Level of science and technology education, logarithm of the proportion of science and technology education in fiscal expenditure	4528	-4.613	0.936
***GTP***	Green technology innovation, measured using the super-efficiency SBM model considering undesirable outputs	4528	0.175	0.095
**ln*ER***	Logarithm of environmental regulation	4528	-10.021	1.290

## 4. Result

### 4.1 Parallel trend test

The parallel trend test is a prerequisite for building a DID model, that is, before the implementation of the NPIB policy, the trends of energy efficiency and carbon emission intensity in pilot cities and non-pilot cities should be similar. Therefore, this study draws on the event analysis method used by Beck et al. [52] and Li et al. [53] to conduct a parallel trend hypothesis test on the implementation group and the control group, and uses 2014 as the base year.

**Fig 2:**
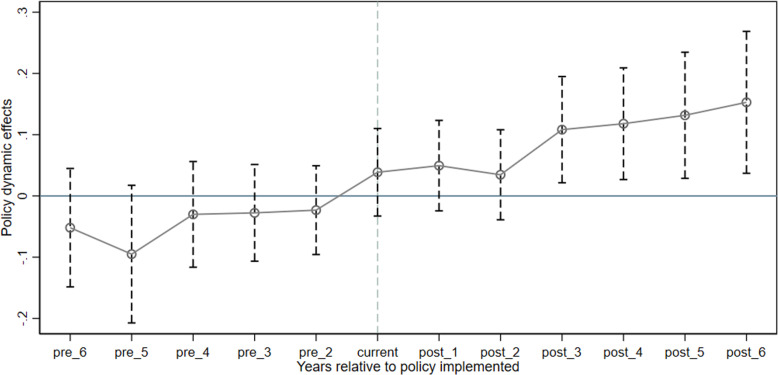
Parallel trend test for energy efficiency.

**Fig 3:**
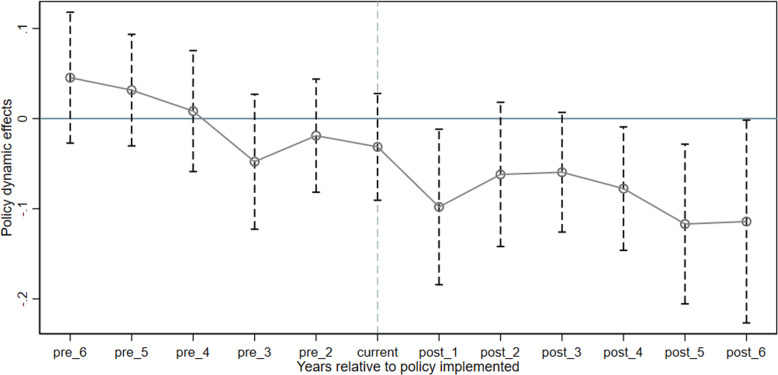
Parallel trend test of carbon emission intensity.

When urban energy efficiency is used as the explained variable, as shown in the parallel trend test results in Fig 2, in each period before the implementation of the NPIB policy, all coefficients failed to pass the statistical test of significance. This shows that, without launching the NPIB policy, there is no significant difference in the energy efficiency of the cities in the experimental group and the control group, and the parallel trend test is passed. In the third year of policy implementation, the estimated coefficient is significantly positive, indicating that after the implementation of the policy, the energy efficiency of the cities in the experimental group is significantly higher than that of the control group. In addition, the estimated coefficient continues to increase from the year after the policy is implemented, indicating that the information-benefiting national pilot project has a long-term time dynamic effect on improving urban energy efficiency, and this effect shows an increasing trend with time. When urban carbon emission intensity is used as the explained variable, as shown in the parallel trend test results in Fig 3, before the policy was implemented, the estimated coefficients of the pilot project were not significant, indicating that before the policy was implemented, the experimental group and the control group cities There are no significant differences in carbon emission intensity and the parallel trend assumption is satisfied. However, after the third year of policy implementation, the estimated coefficients are all significantly negative, indicating that there is a significant difference in the carbon emission intensity of pilot cities and non-pilot cities. That is, the carbon emission intensity of the experimental group cities after 2014 is significantly lower. in the control group. The above verifies that the DID model used in this article is effective.

### 4.2 Regression results

Table 2 reports the empirical test results of digital governance on urban energy conservation and emission reduction. Column (1) reports the regression results when only the explanatory variables and fixed effects are added when the explained variable is urban energy utilization efficiency. The coefficient of the multiplication term *Treat×Post* is 0.1291 and is significant at the 1% statistical level, indicating that the figure Governance significantly improves urban energy efficiency. Column (2) adds other control variables on the basis of column (1). The coefficient of the cross product *Treat×Post* is 0.1209, which is significant at the 1% statistical level. The small change range of the coefficient indicates that the quasi-natural experiment selected in this article has strong exogeneity and is less potentially affected by regional resource endowment factors and other unobservable factors. Columns (3) and (4) of Table 2 report the regression results without adding control variables and after adding control variables respectively when the explained variable is urban carbon emission intensity. The coefficients of the core explanatory variables in these two columns are all negatively significant at the 1% level, and the coefficients do not change significantly, indicating that the implementation of the policy significantly promotes urban carbon emission reduction. Based on the results in column (4), the estimated coefficient of the NPIB policy is -0.0710, which means that when other factors remain unchanged, the pilot project reduced the city's carbon emission intensity by an average of 7.10%. Hypothesis H1 is confirmed.

**Table 2:** Benchmark regression results.

Variables	(1)	(2)	(3)	(4)
	ln*Energy*	ln*Energy*	ln*CO2*	ln*CO2*
*Treat* ***×*** *Post*	0.1469*** (7.14)	0.1252*** (5.85)	-0.1056*** (-6.00)	-0.0645*** (-3.67)
**ln*POP***	－	0.1298 * (1.83)	－	0.0055 (0.07)
***DE***	－	-0.0076 * (-1.69)	－	-0.0058 * (-1.67)
**ln*AIS***	－	-0.2009*** (-7.07)	－	0.1602*** (6.24)
**ln*STE***	－	0.0633*** (4.98)	－	-0.0455*** (-4.44)
***GTP***	－	0.2192** (2.30)	－	-0.3245*** (-3.07)
**ln*ER***	－	0.0182 (1.57)	－	-0.0832*** (-8.69)
***City FE***	Yes	Yes	Yes	Yes
***Year FE***	Yes	Yes	Yes	Yes
*Constant*	2.5109*** (433.74)	2.1612*** (4.77)	-8.4095*** (-1,844.46)	-9.4090*** (-19.48)
*Observations*	4528	4528	4528	4528
*R-squared*	0.823	0.828	0.867	0.873

Notes: Robust t-statistics in parentheses, ***p <  0.01, **p <  0.05, * p <  0.1, respectively.

### 4.3 Robustness test

#### 4.3.1 Placebo test.

Since the benchmark regression results of this paper may have problems with omitted variables and other unobservable factors, this paper adopts the random placebo test method to test whether there are serious measurement errors in the model setting, so as to ensure that the research conclusions of this paper are driven by policy effects rather than random factors. Specifically, this paper randomly selects 71 cities from the sample cities as false treatment group cities, and the remaining 212 cities as false control group cities, keeping the same control variables as formula (1). At the same time, on the basis of controlling the city fixed effect and time fixed effect, the counterfactual test is carried out according to the benchmark regression model, and the coefficient estimate of the impact of the national pilot policy of information benefiting the people on urban energy conservation and emission reduction after the implementation of the city placebo can be obtained. Furthermore, the above process is repeated 1000 times, and finally the kernel density distribution map of the coefficient estimate of the interaction term *Treat×Post* and the kernel density distribution map of the t value are drawn, based on which it is verified whether the energy efficiency and carbon emission intensity of the city are affected by other factors besides the NPIB policy.

**Fig 4:**
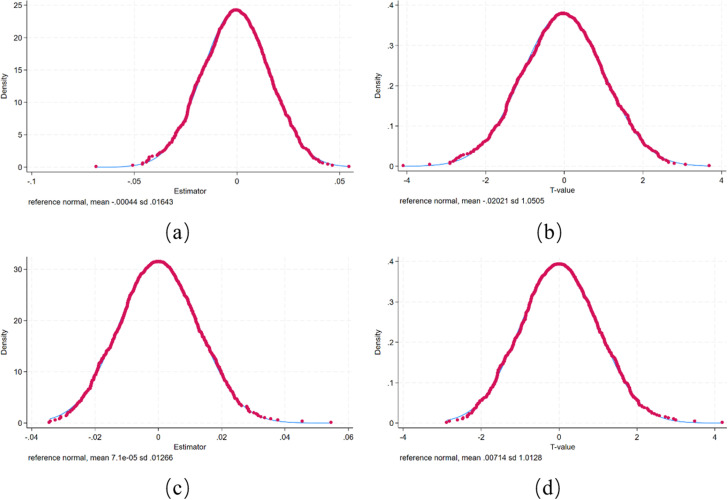
Placebo test results. (a) Kernel density plot of regression coefficients of urban energy efficiency. (b) T-value kernel density map of urban energy efficiency. (c) Regression coefficient kernel density map of urban carbon emission intensity. (d) T-value kernel density map of urban carbon emission intensity.

As shown in Fig 4, the estimated coefficients and t-values of the double difference terms are concentrated around 0, indicating that the urban energy conservation and emission reduction promotion effect identified in the benchmark analysis is indeed the result of the implementation of the pilot policy that this paper focuses on. There is no serious omitted variable problem in the model established in this paper, and the benchmark regression results are reliable.

#### 4.3.2 PSM-DID.

In order to avoid the systematic differences in energy efficiency and carbon emission intensity between cities implementing the NBIP policy and other non-pilot cities, which may cause bias in the regression results of the double difference model, this paper adopts the propensity score matching method (PSM-DID) for robustness test. First, this paper uses the control variables in model (1) to perform *Logit* estimation on the grouping variable *Treat* of the experimental group cities and the control group cities, and uses the predicted value as the score. Secondly, according to the score, k-nearest neighbor matching method (1:4), caliper matching method, Mahalanobis matching method and kernel matching method are used to match the experimental group to the control group with similar characteristics in all aspects. Finally, the matched samples are subjected to DID test according to model (1).

From the regression results of the PSM-DID model (Table 3), it can be found that when urban energy utilization efficiency is used as the explained variable, the regression coefficients of digital governance under various matching methods have passed the significance test at the 1% level.; When urban carbon emission intensity is used as the explained variable, the impact of digital governance on urban carbon emission intensity under various matching methods is still significantly negative at the 1% level. This shows that even after taking into account the bias caused by non-random selection of samples, digital governance can still promote energy conservation and emission reduction in cities. It proves that the results obtained in this paper in the benchmark regression are very robust.

**Table 3:** Results of PSM-DID model.

Variables	k-nearest neighbor		Caliper matching		Mahalanobis matching		Kernel matching	
	(1)	(2)	(3)	(4)	(5)	(6)	(7)	(8)
	ln*Energy*	ln*CO2*	ln*Energy*	ln*CO2*	ln*Energy*	ln*CO2*	ln*Energy*	ln*CO2*
*Treat* ***×*** *Post*	0.1259*** (5.86)	-0.0657*** (-3.74)	0.1259*** (5.86)	-0.0657*** (-3.74)	0.1252*** (5.85)	-0.0645*** (-3.67)	0.1259*** (5.86)	-0.0657*** (-3.74)
***Controls***	Yes	Yes	Yes	Yes	Yes	Yes	Yes	Yes
***City FE***	Yes	Yes	Yes	Yes	Yes	Yes	Yes	Yes
***Year FE***	Yes	Yes	Yes	Yes	Yes	Yes	Yes	Yes
*Constant*	2.1684*** (4.53)	-9.8442*** (-21.76)	2.1684*** (4.53)	-9.8442*** (-21.76)	2.1612*** (4.77)	-9.4090*** (-19.48)	2.1684*** (4.53)	-9.8442*** (-21.76)
*Observations*	4471	4522	4,471	4522	4528	4528	4471	4471
*R-squared*	0.827	0.873	0.827	0.873	0.828	0.873	0.827	0.873

Notes: Robust t-statistics in parentheses, ***p <  0.01, **p <  0.05, * p <  0.1, respectively.

#### 4.3.3 Shrinkage.

In order to avoid outliers in the sample from biasing the benchmark regression results, this paper chooses to perform bilateral winnowing processing on the continuous variables at the 1%, 5% and 10% levels respectively, and re-run the regression on model (1). The results are shown in Table 4. The results after data processing show that the estimated coefficient of the cross-multiplication term *Treat ×  Post* is still significant at the 1%, 5% and 10% level, and its sign is consistent with the benchmark regression results. The robustness of the baseline regression results is again confirmed.

**Table 4:** Wincing results.

Variables	1% tailing treatment		5% tailing treatment		10% tailing treatment	
	(1)	(2)	(3)	(4)	(5)	(6)
	ln*Energy*	ln*CO2*	ln*Energy*	ln*CO2*	ln*Energy*	ln*CO2*
*Treat* ***×*** *Post*	0.1081*** (5.46)	-0.0523*** (-3.06)	0.0991*** (5.47)	-0.0347** (-2.21)	0.0876*** (5.21)	-0.0285** (-1.97)
***Controls***	Yes	Yes	Yes	Yes	Yes	Yes
***City FE***	Yes	Yes	Yes	Yes	Yes	Yes
***Year FE***	Yes	Yes	Yes	Yes	Yes	Yes
*Constant*	1.8856*** (4.56)	10.5788*** (-24.57)	1.8856*** (4.56)	10.2699*** (-22.93)	1.3378*** (3.08)	10.2699*** (-22.93)
*Observations*	4528	4528	4528	4528	4528	4528
*R-squared*	0.834	0.872	0.834	0.865	0.813	0.865

Notes: Robust t-statistics in parentheses, ***p <  0.01, **p <  0.05, * p <  0.1, respectively.

#### 4.3.4 Eliminate other policy interference.

In order to avoid the possible interference of other city-level policies on the estimated results, this paper considers the potential impact of the “National Big Data Comprehensive Experimental Zone Pilot Policy”, “Broadband China Pilot Policy”, and “Smart City Pilot Policy” implemented during the same period, and Sensitivity tests were conducted on sample data before the implementation of these policies. The areas selected for the above pilot policies overlap with the research samples of this article in the time dimension. In order to ensure the purity and accuracy of the research results of this article, this article deletes the overlapping pilot cities located in the above three policies to avoid potential policy interference. As shown in Table 5, even after excluding the city data participating in the construction of the “National Big Data Comprehensive Pilot Zone”, the city data of the “Broadband China Pilot Policy” and the city data of the “Smart City Pilot Policy”, the transportation item *Treat×Post* The regression coefficient of is still significant at the 1% level. This result fully proves that after excluding policy factors, the positive impact of digital governance on urban energy conservation and emission reduction is still stable and has significant practical effects.

**Table 5:** Consider the impact other policies.

Variables	*National Big Data Comprehensive Pilot Zone*		*Broadband China Pilot Policy*		*Smart City Pilot Policy*	
	(1)	(2)	(3)	(4)	(5)	(6)
	ln*Energy*	ln*CO2*	ln*Energy*	ln*CO2*	ln*Energy*	ln*CO2*
*Treat* ***×*** *Post*	0.1115*** (4.60)	-0.0729*** (-3.68)	0.1026*** (3.10)	-0.0527** (-2.03)	0.1553*** (3.63)	-0.0676 * (-1.93)
***Controls***	Yes	Yes	Yes	Yes	Yes	Yes
***City FE***	Yes	Yes	Yes	Yes	Yes	Yes
***Year FE***	Yes	Yes	Yes	Yes	Yes	Yes
*Constant*	2.1787*** (4.27)	-10.0553*** (-20.18)	2.1319*** (3.69)	-9.6178*** (-17.81)	1.4126*** (1.62)	-8.9223*** (-11.12)
*Observations*	3744	3744	3824	3824	2160	2160
*R-squared*	0.824	0.865	0.818	0.857	0.811	0.865

Notes: Robust t-statistics in parentheses, ***p <  0.01, **p <  0.05, * p <  0.1, respectively.

## 5. Further analysis

### 5.1 Mechanism test

The baseline regression results in the previous article show that digital governance has significantly promoted energy conservation and emission reduction in cities. To further reveal its internal mechanism, this paper will explore the mechanism by which digital governance affects urban energy efficiency and carbon emission intensity. This paper introduces green technology innovation as the mediating variable of the energy-saving effect of digital governance and environmental regulation as the mediating variable of the emission reduction effect of digital governance for regression analysis. The specific regression results are detailed in Table 6.

**Table 6:** Mechanism analysis results.

Variable	*GTP*		ln*ER*	
	(1)	(2)	(3)	(4)
***Treat×Post***	0.0365*** (9.43)	0.0291*** (8.11)	0.2164*** (6.52)	0.1410*** (4.19)
***Controls***	No	Yes	No	Yes
***City FE***	Yes	Yes	Yes	Yes
***Year FE***	Yes	Yes	Yes	Yes
***Constant***	0.1703*** (212.72)	-0.2790 (-1.51)	-10.0485*** (-1,215.88)	-10.0842*** (-12.01)
***Observations***	4528	4528	4528	4528
***R-squared***	0.775	0.783	0.868	0.872

Notes: Robust t-statistics in parentheses, ***p <  0.01, **p <  0.05, * p <  0.1, respectively.

#### 5.1.1 The impact of green technology innovation.

In column (1) of Table 6, the explained variable is green technology innovation, and the estimated coefficient of the interaction term Treat×Post is 0.0365, which is significantly positive at the 1% level. After adding the control variables in column (2), the estimated coefficient of the interaction term Treat×Post drops to 0.0291, which is still significantly positive at the 1% level. The regression results show that digital governance can improve urban energy efficiency by promoting green technology innovation.

Government digital management uses digital technology and public open digital platforms to optimize urban resource allocation, improve information transparency, and enhance policy implementation, promote corporate green innovation and investment, and improve economic output efficiency while reducing resource consumption and environmental pollution. Therefore, by promoting green technology progress, digital governance can help achieve urban energy-saving goals, thus confirming hypothesis H2.

#### 5.1.2 The impact of environmental regulation.

In column (3) of Table 6, the explained variable is environmental regulation, and the estimated coefficient of the interaction term Treat×Post is 0.2164, which is significantly positive at the 1% level. After adding the control variables in column (4), the estimated coefficient of the interaction term Treat×Post drops to 0.1410, which is still significantly positive at the 1% level. The regression results show that digital governance can reduce urban carbon emission intensity by strengthening environmental regulation.

Relying on advanced information technology and data analysis methods, based on data-driven decision-making, real-time monitoring, analysis and evaluation of urban energy consumption and pollution emissions can be achieved, so as to formulate and implement refined and targeted environmental regulation measures, improve the accuracy and execution efficiency of environmental regulation, and make regulatory policies more scientific and operational. Therefore, digital governance indirectly promotes the realization of urban emission reduction targets by optimizing the design and implementation of government environmental regulations, thereby verifying hypothesis H3.

### 5.2 Heterogeneity analysis

By conducting various types of robustness tests on the research samples, this paper verifies that digital governance has a promoting effect on energy conservation and emission reduction in cities. However, considering that the effect of digital governance in promoting energy conservation and emission reduction in cities may vary depending on the location, size, and resource status of the city itself. In order to explore the potential diversity of the role of digital governance in promoting energy conservation and emission reduction in cities, this paper examines the changes in the impact of digital governance on promoting energy conservation and emission reduction in cities from the perspectives of geographical location, city size, and urban resource endowment among cities.

#### 5.2.1 Regional heterogeneity.

The effect of digital governance on energy conservation and emission reduction in cities may vary depending on geographical location. Generally speaking, there are significant differences in economic and technological development levels, resource endowment characteristics and policy environment between China’s eastern, central and western regions. In view of these differences, and based on the classification standards for the eastern, central and western regions provided by the National Bureau of Statistics of China, this paper subdivides the research sample into three subsamples in the eastern, central and western regions.

The regional sample regression results in Table 7 show that the effect of digital governance on energy conservation and emission reduction in cities varies from region to region. It is obvious that when energy efficiency is used as the explained variable, the estimated coefficients of digital governance in eastern, central and western China are significantly positive at the 1% level. The difference is that this positive impact varies significantly between different regions, specifically manifested as “eastern <central <western”. The possible reason is that the central and western regions of China as a whole have relatively backward economic and technological development levels, relatively unbalanced resource allocation, and a large proportion of traditional industries. Compared with the developed eastern regions, the introduction of government digital governance can bring more significant improvements in these aspects.

**Table 7:** Regional heterogeneity results.

Variables	ln*Energy*			ln*CO2*		
	(1)	(2)	(3)	(4)	(5)	(6)
	*East*	*Mid*	*West*	*East*	*Mid*	*West*
*Treat* ***×*** *Post*	0.0816*** (2.85)	0.1279*** (2.62)	0.1508*** (4.08)	-0.0737*** (-3.33)	-0.0461 (-1.18)	-0.0446 (-1.51)
***Controls***	Yes	Yes	Yes	Yes	Yes	Yes
***City FE***	Yes	Yes	Yes	Yes	Yes	Yes
***Year FE***	Yes	Yes	Yes	Yes	Yes	Yes
*Constant*	-1.7265 * (-1.82)	3.6632*** (3.00)	1.5208*** (3.04)	-5.9837*** (-6.24)	-10.4977*** (-8.70)	-9.4125*** (-16.30)
*Observations*	1584	1344	1600	1584	1,344	1,600
*R-squared*	0.786	0.841	0.858	0.869	0.857	0.897

Notes: Robust t-statistics in parentheses, ***p <  0.01, **p <  0.05, * p <  0.1, respectively.

According to the empirical results of carbon emission intensity in columns (4), (5) and (6) of Table 7, Digital governance has a significant effect on curbing carbon emissions in eastern cities, but has no significant impact on central and western regions. With its strong economic foundation, improved information infrastructure and technological innovation advantages, the eastern region provides support for green and intelligent development, and helps upgrade low-carbon technologies and improve emission reduction efficiency. However, the central and western regions are limited by their development level. Under the growth model of taking over the transfer of high-energy-consuming industries and relying on heavy industry, they are superimposed with technological shortcomings and insufficient innovation resources, resulting in industrial structural contradictions and multiple obstacles in carbon emission reduction, and the effectiveness of government governance is limited.

#### 5.2.2 Heterogeneity of city size.

The implementation of the “Information Benefiting the People” pilot policy in cities of different sizes may have different effects on the city's energy conservation and emission reduction. This article refers to the city size classification standards published by the State Council in the “Notice of the State Council on Adjusting the Criteria for the Classification of City Scales” (2014), and divides the sample cities into small and medium-sized cities and large cities and above according to the population size of prefecture-level cities. Among them, small and medium-sized cities It refers to cities with a population of no more than 1 million, and large cities and above refers to cities with a population of more than 1 million. Table 8 reports the impact of digital governance on energy conservation and emission reduction in small and medium-sized cities and large cities and above. The regression results by city scale show that the impact coefficients of government digital governance on energy efficiency and carbon emission intensity in large cities and above are 0.2305 and -0.0631 respectively, and both are significant at the 1% level. Digital governance has not had a significant impact on the energy conservation and emission reduction effects of small and medium-sized cities. The possible reason for the city-scale heterogeneity in policy effects is that large cities have relatively complete digital infrastructure, a high degree of informatization, significant human capital advantages, and a good innovation environment. These advantages create an excellent foundation for government digital governance. On the other hand, small cities have shortcomings such as a weak digital technology foundation and a lack of talents, which prevents government digital governance from exerting its due effect.

**Table 8:** Results of city size heterogeneity.

*Variables*	ln*Energy*		ln*CO2*	
	*(1)*	*(2)*	*(3)*	*(4)*
	*Small and medium-sized cities*	*Large cities and above*	*Small and medium-sized cities*	*Large cities and above*
*Treat* ***×*** *Post*	0.1029 (0.67)	0.1210*** (5.48)	-0.1924 (-1.52)	-0.0581*** (-3.31)
***Controls***	Yes	Yes	Yes	Yes
***City FE***	Yes	Yes	Yes	Yes
***Year FE***	Yes	Yes	Yes	Yes
*Constant*	3.8709 * (1.73)	1.9985*** (3.54)	-7.4496*** (-3.08)	-9.0994*** (-15.60)
**Observations**	185	4341	185	4,341
**R-squared**	0.893	0.811	0.910	0.868

Notes: Robust t-statistics in parentheses, ***p <  0.01, **p <  0.05, * p <  0.1, respectively.

#### 5.2.3 Heterogeneity of urban resource endowment.

Resource endowment is an important pillar of urban development. Different resource endowments will lead to differences in economic growth models, information technology and government digital governance levels, which in turn affects the effect of digital governance on promoting urban energy conservation and emission reduction. Based on this, this paper divides 283 sample cities into two types: resource-based cities and non-resource-based cities based on the “National Sustainable Development Plan for Resource-Based Cities (2013–2020)” publicly released by the State Council, and conducts heterogeneity analysis.

Observing columns (1) and (2) of Table 9, we can find that the estimated coefficients of digital governance are 0.1706 and 0.1263 respectively, and both are significantly positive at the 1% level. From this point of view, digital governance promotes the energy efficiency of both resource-based and non-resource-based cities, but the former has a more significant improvement. Resource-based cities are affected by the “resource curse” and have problems such as low energy efficiency and heavy pollution, and there is a lot of room for improvement. Digital governance can effectively break through the traditional extensive development model by optimizing energy consumption monitoring and pollution control, and the marginal effect is easier to show. However, the basic value of energy efficiency in non-resource-based cities is relatively high, and the application of technology has become mature, so the marginal improvement space of digital governance is relatively limited.

**Table 9:** Heterogeneity of urban resource endowment.

Variables	*Energy*		*CO2*	
	*(1)*	*(2)*	*(4)*	*(5)*
	*Resource-based city*	*Non-resource cities*	*Resource-based city*	*Non-resource cities*
*Treat* ***×*** *Post*	0.1706*** (3.57)	0.1263*** (4.86)	0.0625 (1.65)	-0.1385*** (-6.50)
***Controls***	Yes	Yes	Yes	Yes
***City FE***	Yes	Yes	Yes	Yes
***Year FE***	Yes	Yes	Yes	Yes
*Constant*	1.5643*** (2.62)	2.1604*** (3.13)	-9.1809*** (-15.15)	-9.5846*** (-13.72)
*Observations*	1824	2704	1824	2704
*R-squared*	0.836	0.826	0.859	0.845

Notes: Robust t-statistics in parentheses, ***p <  0.01, **p <  0.05, * p <  0.1, respectively.

The group regression results in columns (3) and (4) of Table 9 show that digital governance significantly promotes carbon emission reduction in non-resource-based cities, but has no significant impact on the carbon emission intensity of resource-based cities. The possible reason is that the economy of resource-based cities is highly dependent on resource extraction and primary processing industries with high energy consumption and high carbon emissions, resulting in long-term high carbon emission levels and greater difficulty in achieving carbon emission reductions. In contrast, the economic structure of non-resource-based cities is relatively diversified, and digital governance can more effectively implement low-carbon measures in areas such as transportation, buildings, and services, thus achieving significant results in carbon emission reduction.

## 6. Conclusions and recommendations

### 6.1 Conclusions

This study uses panel data from 283 cities in China from 2006 to 2021 to conduct an empirical study on the effect of digital governance in influencing urban energy conservation and emission reduction. According to the research, digital governance has achieved remarkable results in promoting urban energy conservation and emission reduction. Among them, the impact on energy efficiency is more significant in the western region, large-scale cities and resource-based cities with resources as the main source of economic growth, while the impact on carbon emission reduction is more significant in the eastern region, large-scale cities and non-resource-based urban areas. We also used various methods to conduct robustness tests, such as placebo tests, PSM-DID, excluding other policy interferences, and shrinking the samples. These tests ensure the reliability and stability of the estimated results. In addition, the energy conservation and emission reduction effects of digital governance can be further enhanced through green technology innovation and environmental regulation.

### 6.2 Recommendations

Based on the above research results, we provide the following countermeasures and suggestions:

First, improve the digital governance policy system. The government should strengthen policy support for digital governance, formulate practical and forward-looking governance plans based on regional needs, characteristics and resource endowments, promote the effective implementation of policies, and achieve efficient government governance and green and sustainable urban development in the digital age.

Second, the government should promote the application of digital technology in energy management and pollution control, optimize resource allocation and improve energy efficiency, build a sustainable urban ecology, and achieve a win-win situation for the economy and the environment.

Third, further strengthen environmental regulation. In light of the actual situation of urban development, timely adjust environmental regulatory policies, increase the diversity and flexibility of regulatory forms, such as establishing carbon emission statistical accounting and green tax systems, to ensure the effective implementation of environmental regulatory policies and achieve green and high-quality urban development.

## Supporting information

S1 File Data.
(XLSX)

S2 File Order.
(DOCX)

## References

[1] Ebi KL, Vanos J, Baldwin JW, Bell JE, Hondula DM, Errett NA, et al. Extreme Weather and Climate Change: Population Health and Health System Implications. Annu Rev Public Health. 2021;42:293–315. doi: 10.1146/annurev-publhealth-012420-105026 33406378 PMC9013542

[2] Böhringer C, Peterson S, Rutherford TF, Schneider J, Winkler M. Climate policies after paris: Pledge, trade and recycle: Insights from the 36th energy modeling forum study (emf36). Energy Economics. 2021;103:105471.

[3] Chen XH, Tee K, Elnahass M, Ahmed R. Assessing the environmental impacts of renewable energy sources: A case study on air pollution and carbon emissions in China. J Environ Manage. 2023;345:118525. doi: 10.1016/j.jenvman.2023.118525 37421726

[4] Sun Y, Zhou C. Which works better? Comparing the multiple effects of heterogeneous environmental regulations on urban green economic transformation in China. J Environ Manage. 2024;368:122124. doi: 10.1016/j.jenvman.2024.122124 39126847

[5] Wang F, Wu M. How does trade policy uncertainty affect China’s economy and energy?. J Environ Manage. 2023;330:117198. doi: 10.1016/j.jenvman.2022.117198 36603270

[6] Song X, Yao Y, Wu X. Digital finance, technological innovation, and carbon dioxide emissions. Economic Analysis and Policy. 2023;80:482–94. doi: 10.1016/j.eap.2023.09.005

[7] Xu T, Kang C, Zhang H. China’s efforts towards carbon neutrality: Does energy-saving and emission-reduction policy mitigate carbon emissions?. J Environ Manage. 2022;316:115286. doi: 10.1016/j.jenvman.2022.115286 35658256

[8] Feng T, Lin Z, Du H, Qiu Y, Zuo J. Does low-carbon pilot city program reduce carbon intensity? Evidence from Chinese cities. Research in International Business and Finance. 2021;58:101450. doi: 10.1016/j.ribaf.2021.101450

[9] Guo Q, Wang Y, Dong X. Effects of smart city construction on energy saving and CO2 emission reduction: Evidence from China. Applied Energy. 2022;313:118879.

[10] Xu Q, Zhong M, Li X. How does digitalization affect energy? International evidence. Energy Economics. 2022;107:105879.

[11] Broccardo L, Zicari A, Jabeen F, Bhatti ZA. How digitalization supports a sustainable business model: A literature review. Technological Forecasting and Social Change. 2023;187:122146. doi: 10.1016/j.techfore.2022.122146

[12] Zhao S, Teng L, Arkorful VE, Hu H. Impacts of digital government on regional eco-innovation: Moderating role of dual environmental regulations. Technological Forecasting and Social Change. 2023;196:122842. doi: 10.1016/j.techfore.2023.122842

[13] Chen K, Li Q, Shoaib M, Ameer W, Jiang T. Does improved digital governance in government promote natural resource management? Quasi-natural experiments based on smart city pilots. Resources Policy. 2024;90:104721. doi: 10.1016/j.resourpol.2024.104721

[14] Shahbaz M, Wang J, Dong K, Zhao J. The impact of digital economy on energy transition across the globe: The mediating role of government governance. Renewable and Sustainable Energy Reviews. 2022;166:112620. doi: 10.1016/j.rser.2022.112620

[15] Iyke BN. Climate change, energy security risk, and clean energy investment. Energy Economics. 2024;129 107225. doi: 10.1016/j.eneco.2023.107225

[16] Rahman A, Murad SMW, Mohsin AKM, Wang X. Does renewable energy proactively contribute to mitigating carbon emissions in major fossil fuels consuming countries?. Journal of Cleaner Production. 2024;452:142113. doi: 10.1016/j.jclepro.2024.142113

[17] Tan L, Yang Z, Irfan M, Ding CJ, Hu M, Hu J. Toward low‐carbon sustainable development: Exploring the impact of digital economy development and industrial restructuring. Bus Strat Env. 2023;33(3):2159–72. doi: 10.1002/bse.3584

[18] Wang Y, Zhao Z, Shi M, Liu J, Tan Z. Public environmental concern, government environmental regulation and urban carbon emission reduction-Analyzing the regulating role of green finance and industrial agglomeration. Sci Total Environ. 2024;924:171549. doi: 10.1016/j.scitotenv.2024.171549 38467260

[19] Li H, Zhang Y, Li Y. The impact of the digital economy on the total factor productivity of manufacturing firms: Empirical evidence from China. Technological Forecasting and Social Change. 2024;207:123604. doi: 10.1016/j.techfore.2024.123604

[20] Zahra SA, Liu W, Si S. How digital technology promotes entrepreneurship in ecosystems. Technovation. 2023;119:102457. doi: 10.1016/j.technovation.2022.102457

[21] Xu Q, Zhong M, Dong Y. Digital finance and rural revitalization: Empirical test and mechanism discussion. Technological Forecasting and Social Change. 2024;201:123248.

[22] Engås KG, Raja JZ, Neufang IF. Decoding technological frames: An exploratory study of access to and meaningful engagement with digital technologies in agriculture. Technological Forecasting and Social Change. 2023;190:122405. doi: 10.1016/j.techfore.2023.122405

[23] Liu Y, Zhang X, Shen Y. Technology-driven carbon reduction: analyzing the impact of digital technology on China’s carbon emission and its mechanism. Technological Forecasting and Social Change. 2024;200:123124.

[24] Zou S, Liao Z, Fan X. The impact of the digital economy on urban total factor productivity: mechanisms and spatial spillover effects. *Scientific Reports.* 2024;14(1): 396. 38172495 10.1038/s41598-023-49915-3 PMC10764890

[25] Yang X, Ran R, Chen Y, Zhang J. Does digital government transformation drive regional green innovation? Evidence from cities in China. Energy Policy. 2024;187:114017.

[26] Wei X, Jiang F, Yang L. Does digital dividend matter in China’s green low-carbon development: Environmental impact assessment of the big data comprehensive pilot zones policy. Environmental Impact Assessment Review. 2023;101:107143.

[27] Dong F, Li Y, Qin C, Zhang X, Chen Y, Zhao X, et al. Information infrastructure and greenhouse gas emission performance in urban China: A difference-in-differences analysis. J Environ Manage. 2022;316:115252. doi: 10.1016/j.jenvman.2022.115252 35594820

[28] Yang Z, Gao W, Han Q, Qi L, Cui Y, Chen Y. Digitalization and carbon emissions: How does digital city construction affect China's carbon emission reduction?. Sustainable Cities and Society. 2022;87:104201. doi: 10.1016/j.scs.2022.104201

[29] Dian J, Song T, Li S. Facilitating or inhibiting? Spatial effects of the digital economy affecting urban green technology innovation. Energy Economics. 2024;129:107223. doi: 10.1016/j.eneco.2023.107223

[30] Dong K, Yang S, Wang J. How digital economy lead to low-carbon development in China? The case of e-commerce city pilot reform. Journal of Cleaner Production. 2023;391:136177. doi: 10.1016/j.jclepro.2023.136177

[31] Gu B, Liu J, Ji Q. The effect of social sphere digitalization on green total factor productivity in China: Evidence from a dynamic spatial Durbin model. J Environ Manage. 2022;320:115946. doi: 10.1016/j.jenvman.2022.115946 35961145

[32] ElMassah S, Mohieldin M. Digital transformation and localizing the Sustainable Development Goals (SDGs). Ecological Economics. 2020;169:106490. doi: 10.1016/j.ecolecon.2019.106490

[33] Chen X, Wang J. Unleashing the power of informatization: How does the “information benefiting people” policy affect green total factor productivity?. J Environ Manage. 2023;341:118083. doi: 10.1016/j.jenvman.2023.118083 37150172

[34] Wang H, Li Y, Lin W, Wei W. How does digital technology promote carbon emission reduction? Empirical evidence based on e-commerce pilot city policy in China. J Environ Manage. 2023;325(Pt A):116524. doi: 10.1016/j.jenvman.2022.116524 36272294

[35] Hu J. Synergistic effect of pollution reduction and carbon emission mitigation in the digital economy. J Environ Manage. 2023;337:117755. doi: 10.1016/j.jenvman.2023.117755 36948146

[36] Shang Y, Raza SA, Huo Z, Shahzad U, Zhao X. Does enterprise digital transformation contribute to the carbon emission reduction? Micro-level evidence from China. International Review of Economics & Finance. 2023;86:1–13. doi: 10.1016/j.iref.2023.02.019

[37] Li G, Wu H, Jiang J, Zong Q. Digital finance and the low-carbon energy transition (LCET) from the perspective of capital-biased technical progress. Energy Economics. 2023;120:106623. doi: 10.1016/j.eneco.2023.106623

[38] Chang H, Ding Q, Zhao W, Hou N, Liu W. The digital economy, industrial structure upgrading, and carbon emission intensity —— empirical evidence from China’s provinces. Energy Strategy Reviews. 2023;50:101218. doi: 10.1016/j.esr.2023.101218

[39] Luo Y, Cui H, Zhong H, Wei C. Business environment and enterprise digital transformation. Finance Research Letters. 2023;57:104250. doi: 10.1016/j.frl.2023.104250

[40] Dong F, Hu M, Gao Y, Liu Y, Zhu J, Pan Y. How does digital economy affect carbon emissions? Evidence from global 60 countries. Sci Total Environ. 2022;852:158401. doi: 10.1016/j.scitotenv.2022.158401 36057304

[41] Harsono I, Suprapti IAP. The Role of Fintech in Transforming Traditional Financial Services. COUNT. 2024;1(1):81–91. doi: 10.62207/gfzvtd24

[42] Zhang J, Liang F, Gao P. Can big data reduce urban environmental pollution? Evidence from China’s digital technology experimental zone. PLoS One. 2024;19(10):e0288429. doi: 10.1371/journal.pone.0288429 39413142 PMC11482696

[43] Lyu Y, Zhang J, Wang W, Li Y, Geng Y. Toward low carbon development through digital economy: A new perspective of factor market distortion. Technological Forecasting and Social Change. 2024;208:123685. doi: 10.1016/j.techfore.2024.123685

[44] Zou S, Fan X, Zhou Y, et al. Achieving collaborative pollutant and carbon emissions reduction through digital governance: Evidence from Chinese enterprises. *Environmental Research.* 2024. 263: 120197. 39427948 10.1016/j.envres.2024.120197

[45] Zheng Z, Shafique M, Luo X, Wang S. A systematic review towards integrative energy management of smart grids and urban energy systems. Renewable and Sustainable Energy Reviews. 2024;189:114023. doi: 10.1016/j.rser.2024.114023

[46] Lu Q, Fang H, Hou J. The impact of energy supply side on the diffusion of low-carbon transformation on energy demand side under low-carbon policies in China. Energy. 2024;307:132817. doi: 10.1016/j.energy.2024.132817

[47] Feroz AK, Zo H, Chiravuri A. Digital transformation and environmental sustainability: A review and research agenda. Sustainability. 2021;13(3):1530.

[48] Jin Y, Li X, Zeng H, Cheng X. Does digital government transformation inhibit corporate environmental violations? Evidence from the big data bureau in China. IEEE Transactions on Engineering Management. 2023

[49] Xu B. Environmental regulations, technological innovation, and low carbon transformation: A case of the logistics industry in China. Journal of Cleaner Production. 2024;439:140710. doi: 10.1016/j.jclepro.2024.140710

[50] Shi B, Li N, Gao Q, Li G. Market incentives, carbon quota allocation and carbon emission reduction: Evidence from China’s carbon trading pilot policy. J Environ Manage. 2022;319:115650. doi: 10.1016/j.jenvman.2022.115650 35820308

[51] Ma X, Wang C, Yu Y, Li Y, Dong B, Zhang X, et al. Ecological efficiency in China and its influencing factors-a super-efficient SBM metafrontier-Malmquist-Tobit model study. Environ Sci Pollut Res Int. 2018;25(21):20880–98. doi: 10.1007/s11356-018-1949-7 29766421

[52] Beck T, Levine R, Levkov A. Big Bad Banks? The Winners and Losers from Bank Deregulation in the United States. The Journal of Finance. 2010;65(5):1637–67. doi: 10.1111/j.1540-6261.2010.01589.x

[53] Li P, Lu Y, Wang J. Does flattening government improve economic performance? Evidence from China. Journal of development economics. 2016;123:18–37.

