# Supplementary material for: Can digital governance promote urban energy conservation and emission reduction? A quasi-natural experiment based on “National Pilot Policy of Information Benefiting the People” in China
Source: PLoS One. 2025 Mar 25;20(3):e0320007. doi: 10.1371/journal.pone.0320007 (PMC11936266; doi:10.1371/journal.pone.0320007)
Supplement: S2 File — (DOCX) [file pone.0320007.s002.docx]

# Order

clear all

use data

xtset id year

///////////////////////Descriptive statistics///////////////////////////////

sum

outreg2 using 描述性统计.doc, replace sum(log) title(Decriptive statistics)

//////////////////Parallel trend test///////////////////////////////////////////

gen did = treat*post

gen policy = year - 2014

tab policy

replace policy = -5 if policy < -5

replace policy = 7 if policy > 7

forvalues i = 6(-1)1{

gen pre_`i' = (policy == -`i' & treat == 1)

}

gen current = (policy == 0 & treat == 1)

forvalues j = 1(1)6{

gen post_`j' = (policy == `j' & treat == 1)

}

drop pre_1

//////////////////////////////////////////////////////////lnenergy

reghdfe lnenergy did, absorb(id year) vce(r)

reghdfe lnenergy did lnpop de lnais lnste gtp lner, absorb(id year) vce(r)

reghdfe lnnx2 pre_* current post_* , absorb(id year) vce(r)

coefplot, baselevels

keep(pre_* current post_*)

vertical

yline(0,lcolor(edkblue*0.8))

xline(7, lwidth(vthin) lpattern(dash) lcolor(teal))

ylabel(,labsize(*0.75)) xlabel(,labsize(*0.75))

ytitle("Policy dynamic effects", size(small))

xtitle("Years relative to policy implemented", size(small)

addplot(line @b @at)

ciopts(lpattern(dash) recast(rcap) msize(medium))

msymbol(circle_hollow)

scheme(s1mono)

///////////////////////////////////////////////////////////lnco2

reghdfe lnco2 did, absorb(id year) vce(r)

reghdfe lnco2 did lnpop de lnais lnste gtp lner, absorb(id year) vce(r)

reghdfe lnco2 pre_* current post_* , absorb(id year) vce(r)

coefplot, baselevels

keep(pre_* current post_*)

vertical

yline(0,lcolor(edkblue*0.8))

xline(7, lwidth(vthin) lpattern(dash) lcolor(teal))

ylabel(,labsize(*0.75)) xlabel(,labsize(*0.75))

ytitle("Policy dynamic effects", size(small))

xtitle("Years relative to policy implemented", size(small))

addplot(line @b @at)

ciopts(lpattern(dash) recast(rcap) msize(medium))

msymbol(circle_hollow)

scheme(s1mono)

//////////////////Parallel trend test///////////////////////////////////////////

//////////////////Benchmark regression//////////////////////////////////////////

reghdfe lnenergy did, absorb(id year) vce(r)

outreg2 using regression1.doc,tstat bdec(4) tdec(2) keep(lnenergy did) addtext(City FE, YES, Year FE, YES) replace

reghdfe lnenergy did lnpop de lnais lnste gtp lner, absorb(id year) vce(r)

outreg2 using regression2.doc,tstat bdec(4) tdec(2) keep(lnenergy did lnpop de lnais lnste gtp lner) addtext(City FE, YES, Year FE, YES) replace

reghdfe lnco2 did, absorb(id year) vce(r)

outreg2 using regression3.doc,tstat bdec(4) tdec(2) keep(lnco2 did) addtext(City FE, YES, Year FE, YES) replace

reghdfe lnco2 did lnpop de lnais lnste gtp lner, absorb(id year) vce(r)

outreg2 using regression4.doc,tstat bdec(4) tdec(2) keep(lnco2 did lnpop de lnais lnste gtp lner) addtext(City FE, YES, Year FE, YES) replace

//////////////////Benchmark regression//////////////////////////////////////////

///////////////////Robustness test//////////////////////////////////////////////

/////////////////Placebo test///////////////

reghdfe lnenergy did lnpop de lnais lnste gtp lner, absorb(id year) vce(r)

cap erase "simulations.dta"

permute did beta = _b[did] se = _se[did] df = e(df_r), reps(1000) rseed(777) saving("simulations.dta"): reghdfe lnenergy did lnpop de lnais lnste gtp lner, absorb(id year) vce(r)

use "simulations.dta", clear

gen t_value = beta / se

gen p_value = 2 * ttail(df, abs(beta/se))

dpplot beta, xline(.1227, lc(red*0.5) lp(dash)) xtitle("Estimator") ytitle("Density")

dpplot t_value, xtitle("T-value") ytitle("Density")

twoway (scatter p_value beta)(kdensity beta, yaxis(2))

twoway (scatter p_value beta)(kdensity beta)

reghdfe lnco2 did lnpop de lnais lnste gtp lner, absorb(id year) vce(r)

cap erase "simulations.dta"

permute did beta = _b[did] se = _se[did] df = e(df_r), reps(1000) rseed(777) saving("simulations.dta"): reghdfe lnco2 did lnpop de lnais lnste gtp lner, absorb(id year) vce(r)

use "simulations.dta", clear

gen t_value = beta / se

gen p_value = 2 * ttail(df, abs(beta/se))

dpplot beta, xline(-0.0722, lc(red*0.5) lp(dash)) xtitle("Estimator") ytitle("Density")

dpplot t_value, xtitle("T-value") ytitle("Density")

twoway (scatter p_value beta)(kdensity beta, yaxis(2))

twoway (scatter p_value beta)(kdensity beta)

///////////////PSM-DID/////////////////////////

/////lnenergy

logistic treat lnenergy lnpop de lnais lnste gtp lner,coef

predict p

gen q = log((1-p)/p)

set seed 000001

g tmp = runiform()

sort tmp

//k-nearest neighbor（1:4）

psmatch2 treat lnpop de lnais lnste gtp lner, outcome(lnenergy) neighbor(4) common odds logit ties ate

//Caliper matching

psmatch2 treat lnenergy lnpop de lnais lnste gtp lner, outcome(lnenergy) radius caliper(0.2288) logit common ties ate quietly

//Kernel matching

psmatch2 treat lnenergy lnpop de lnais lnste gtp lner, outcome(lnenergy) kernel ate ties logit common quietly

//Mahalanobis matching

psmatch2 treat ,mahalanobis(lnenergy lnpop de lnais lnste gtp lner) outcome(lnenergy) neighbor(4) ai(4) ate

gen common = _support

drop if common == 0

reghdfe lnenergy did lnpop de lnais lnste gtp lner, absorb(id year) vce(r)

outreg2 using PSM-DID1.doc,tstat bdec(4) tdec(2) keep(lnenergy did lnpop de lnais lnste gtp lner) addtext(City FE, YES, Year FE, YES) replace

/////lnco2

logistic treat lnco2 lnpop de lnais lnste gtp lner, coef

predict p

gen q = log((1-p)/p)

set seed 000001

g tmp = runiform()

sort tmp

//k-nearest neighbor（1:4）

psmatch2 treat lnco2 lnpop de lnais lnste gtp lner,outcome(lnco2) neighbor(4) common odds logit ties ate

//Caliper matching

psmatch2 treat lnco2 lnpop de lnais lnste gtp lner, outcome(lnco2) radius caliper(0.2) logit common ties ate quietly

//Kernel matching

psmatch2 treat lnco2 lnpop de lnais lnste gtp lner, outcome(lnco2) kernel ate ties logit common quietly

//Mahalanobis matching

psmatch2 treat, mahalanobis(lnco2 lnpop de lnais lnste gtp lner) outcome(lnco2) neighbor(4) ai(4) ate

gen common = _support

drop if common == 0

reghdfe lnco2 did lnpop de lnais lnste gtp lner, absorb(id year) vce(r)

outreg2 using PSM-DID2.doc,tstat bdec(4) tdec(2) keep(lnco2 did lnpop de lnais lnste gtp lner) addtext(City FE, YES, Year FE, YES) replace

///////////////Shrinkage///////////////////////

//1%

winsor2 lnenergy lnco2 lnpop de lnais lnste gtp lner, cuts(1 99)

//5%

winsor2 lnenergy lnco2 lnpop de lnais lnste gtp lner, cuts(5 95)

//10%

winsor2 lnenergy lnco2 lnpop de lnais lnste gtp lner, cuts(10 90)

reghdfe lnenergy_w did, absorb(id year) vce(r)

outreg2 using Shrinkage1.doc,tstat bdec(4) tdec(2) keep(lnnegrgy did) addtext(Industry FE, YES, Year FE, YES) replace

reghdfe lnenergy_w did lnpop_w de_w lnais_w lnste_w gtp_w lner_w, absorb(id year) vce(r)

outreg2 using Shrinkage2.doc,tstat bdec(4) tdec(2) keep(lnenergy_w did lnpop_w de_w lnais_w lnste_w gtp_w lner_w) addtext(Industry FE, YES, Year FE, YES) replace

reghdfe lnco2_w did, absorb(id year) vce(r)

outreg2 using Shrinkage3.doc,tstat bdec(4) tdec(2) keep(lnco2_w did) addtext(Industry FE, YES, Year FE, YES) replace

reghdfe lnco2_w did lnpop_w de_w lnais_w lnste_w gtp_w lner_w, absorb(id year) vce(r)

outreg2 using Shrinkage4.doc,tstat bdec(4) tdec(2) keep(lnco2_w did lnpop_w de_w lnais_w lnste_w gtp_w lner_w) addtext(Industry FE, YES, Year FE, YES) replace

//////Eliminate other policy interference//////////

reghdfe lnenergy did lnpop de lnais lnste gtp lner if bigdata == 0, absorb(id year) vce(r)

reghdfe lnco2 did lnpop de lnais lnste gtp lner if bigdata == 0, absorb(id year) vce(r)

reghdfe lnenergy did lnpop de lnais lnste gtp lner if broadband == 0, absorb(id year) vce(r)

reghdfe lnco2 did lnpop de lnais lnste gtp lner if broadband == 0, absorb(id year) vce(r)

reghdfe lnenergy did lnpop de lnais lnste gtp lner if smart == 0, absorb(id year) vce(r)

reghdfe lnco2 did lnpop de lnais lnste gtp lner if snart == 0, absorb(id year) vce(r)

///////////////////Robustness test//////////////////////////////////////////////

////////////////////////////Mechanism Test/////////////////////////////////

reghdfe gtp did lnpop de lnais lnste lner, absorb(id year) vce(r)

outreg2 using Mechanism Test1.doc,tstat bdec(4) tdec(2) keep(gtp did lnpop de lnais lnste lner) addtext(City FE, YES, Year FE, YES) replace

reghdfe lner did lnpop de lnais lnste gtp, absorb(id year) vce(r)

outreg2 using Mechanism Test2.doc,tstat bdec(4) tdec(2) keep(lner did lnpop de lnais lnste gtp) addtext(City FE, YES, Year FE, YES) replace

////////////////////////////Mechanism Test/////////////////////////////////

//////////////////Heterogeneity analysis/////////////////////////////////////

//////////////////Regional heterogeneity////////////////

reghdfe lnenergy did lnpop de lnais lnste gtp lner if east == 1, absorb(id year) vce(r)

reghdfe lnenergy did lnpop de lnais lnste gtp lner if mid == 1, absorb(id year) vce(r)

reghdfe lnenergy did lnpop de lnais lnste gtp lner if west == 1, absorb(id year) vce(r)

outreg2 using Regional heterogeneity1.doc,tstat bdec(4) tdec(2) keep(lnenergy did lnpop de lnais lnste gtp lner) addtext(City FE, YES, Year FE, YES) append

reghdfe lnco2 did lnpop de lnais lnste gtp lner if east == 1, absorb(id year) vce(r)

reghdfe lnco2 did lnpop de lnais lnste gtp lner if mid == 1, absorb(id year) vce(r)

reghdfe lnco2 did lnpop de lnais lnste gtp lner if west == 1, absorb(id year) vce(r)

outreg2 using Regional heterogeneity2.doc,tstat bdec(4) tdec(2) keep(lnco2 did lnpop de lnais lnste gtp lner) addtext(City FE, YES, Year FE, YES) append

/////////////////// Heterogeneity of city size////////////

gen scale=1 if pop<100

replace scale=2 if pop>=100

reghdfe lnenergy did lnpop de lnais lnste gtp lner if scale == 1, absorb(id year) vce(r)

reghdfe lnenergy did lnpop de lnais lnste gtp lner if scale == 2, absorb(id year) vce(r)

outreg2 using Heterogeneity of city size1.doc,tstat bdec(4) tdec(2) keep(lnnx2 did lner lnkj lnpop de lnais gtfp) addtext(City FE, YES, Year FE, YES) append

reghdfe lnco2 did lnpop de lnais lnste gtp lner if scale == 1, absorb(id year) vce(r)

reghdfe lnco2 did lnpop de lnais lnste gtp lner if scale == 2, absorb(id year) vce(r)

outreg2 using Heterogeneity of city size2.doc,tstat bdec(4) tdec(2) keep(lnco21 did lner lnkj lnpop de lnais gtfp) addtext(City FE, YES, Year FE, YES) append

/////////////Heterogeneity of urban resource endowment///////

reghdfe lnenergy did lnpop de lnais lnste gtp lner if resource == 1, absorb(id year) vce(r)

reghdfe lnenergy did lnpop de lnais lnste gtp lner if resource == 0, absorb(id year) vce(r)

outreg2 using resource1.doc,tstat bdec(4) tdec(2) keep(lnnx2 did lner lnkj lnpop de lnais gtfp) addtext(City FE, YES, Year FE, YES) append

reghdfe lnco2 did lnpop de lnais lnste gtp lner if resource == 1, absorb(id year) vce(r)

reghdfe lnco2 did lnpop de lnais lnste gtp lner if resource == 0, absorb(id year) vce(r)

outreg2 using resource2.doc,tstat bdec(4) tdec(2) keep(lnco21 did lner lnkj lnpop de lnais gtfp) addtext(City FE, YES, Year FE, YES) append

//////////////////Heterogeneity analysis/////////////////////////////////////
